# Remote data collection using multiple modalities: Successes, challenges, and lessons learned across two randomized controlled trials

**DOI:** 10.1002/alz70860_107323

**Published:** 2025-12-23

**Authors:** Lesley A. Ross, Alyssa A. Gamaldo, Hye Won Chai, Abigail Stephan, Martin J. Sliwinski, Christine Phillips

**Affiliations:** ^1^ Clemson University, Institute for Engaged Aging, Seneca, SC, USA; ^2^ Pennsylvania State University, State College, PA, USA

## Abstract

**Background:**

Remote data collection offers opportunities to reach underserved populations with mobility, time, and access restrictions to large data collection sites. Use of multiple remote data collection modalities ranging from passive (e.g., smartwatches, cameras, etc.) to active (e.g., smartphones, tablets, etc.) technologies also enhance the richness, and potentially accuracy, of data collected. This study presents the design, methodology, and compliance of two randomized controlled clinical trials which utilized multiple remote data collection methods, highlighting the successes and challenges.

**Methods:**

The Everyday Function Trial (EFIT; NCT04651582; *n* = 96) and the Elucidating the Necessary Active Components of Training (ENACT; NCT05366023, *n* = 286) examine moderators and mechanisms underlying cognitive training effects to complex everyday functioning. Data collection included cognitive, lifestyle, psychosocial, health and biomarker measures collected across 6‐9 months. Participants ranged from 55‐85 years of age and completed between 6 and 9 hours of cognitive, psychosocial, health and everyday function assessments on study‐provided laptops; daily cognitive, health and psychosocial assessments on study‐provided smartphones (ecological momentary assessment; EMA), and 0‐40 hours of cognitive training. Subsamples of participants also provided blood samples and/or completed fMRI, sleep assessments, and passive assessments via a smartwatch.

**Results:**

Attrition was relatively low across the studies ranging from 15%‐29%. Compliance varied across study conditions but was generally high (>90%) for both laptop assessments and daily EMA. Missing data were generally due to technical issues rather than participant refusal. The most common reasons for attrition were due to time commitment, dislike of intervention, and technological difficulties.

**Conclusions:**

These studies demonstrate the feasibility of remote data collection. Participant and staff feedback from the studies provide key areas of improvement for future work. Given increasing interest in including middle‐aged adults in Alzheimer's disease and related dementia early identification and prevention, remote methods provide an additional tool for both recruitment and retention of these individuals – especially for complex protocols. Future directions and areas of improvement will be discussed.

